# Facile Fabrication of Attapulgite-Modified Chitosan Composite Aerogels with Enhanced Mechanical Strength and Flame Retardancy for Thermal Insulation

**DOI:** 10.3390/polym18010098

**Published:** 2025-12-29

**Authors:** Siyuan Cheng, Yuwen Shao, Meisi Chen, Chenfei Wang, Xinbao Zhu, Xiongfei Zhang, Bo Fu

**Affiliations:** 1 Jiangsu Co-Innovation Center of Efficient Processing and Utilization of Forest Resources, Huaian Industrial Research Institute, Gas Separation Engineering Technology Research Center, College of Chemical Engineering, Nanjing Forestry University, Nanjing 210037, China; 2 Synorm Chemical Co., Ltd., No. 16 Zijin Road, Circular Economy Zone, Huizhou District, Huangshan 245900, China

**Keywords:** chitosan, aerogel, flame retardant mechanism

## Abstract

Aerogels are recognized as exceptional thermal insulation materials, but poor mechanical strength and flammability problems hinder their application in high-temperature environments. Thermal management materials that combine high mechanical strength with superior flame retardancy are, therefore, critically important for thermal insulation. Herein, ultra-lightweight aerogels were facilely fabricated using chitosan (CS) and acidified attapulgite (SATP) as the primary components. The optimal composite, CS-SATP30%, exhibited a compressive strength of 633.15 kPa at 80% strain, demonstrating significant improvement in mechanical properties. Structural analysis revealed that the hydroxyl groups and amino groups of CS molecules formed hydrogen bonds with SATP, ensuring excellent interfacial affinity among the constituents. Compared to pure CS aerogel, the total heat release (THR) and peak heat release rate (PHRR) of CS-SATP30% were substantially reduced to 3.83 MJ/m^2^ and 37.00 kW/m^2^, respectively. Furthermore, the limiting oxygen index (LOI) of CS-SATP30% increased to 34% and passed the vertical burning test (UL-94). This study provides a feasible way to construct advanced chitosan-based thermal insulation aerogels.

## 1. Introduction

The rapid advancement of industrialization and urbanization has resulted in a relentless escalation of energy consumption, posing a significant challenge to sustainable development [1,2,3]. Currently, the development and utilization of efficient thermal insulators are widely regarded as a direct and effective strategy for saving energy and reducing carbon emissions [4]. Regarding thermal insulation materials, inorganic options, such as mineral wool, often exhibit poor mechanical properties and unsatisfactory thermal performance [5,6]. In contrast, although organic insulating materials, like epoxy resin, offer good thermal insulation, their preparation processes can cause environmental pollution, leading to their declining popularity [7,8].

Aerogels, among the lightest known solid materials, have already been used in diverse fields as a thermal insulating material because of their low density, high porosity, and relatively low thermal conductivity [9,10,11]. Unlike conventional petroleum-based organic insulation materials, aerogels derived from natural polysaccharides offer distinct advantages such as wide availability, eco-friendliness, and biodegradability [12,13]. As a representative bio-based aerogel, chitosan aerogels are promising for thermal insulation owing to their structural plasticity and the intrinsic flame retardant potential associated with their nitrogen content [14,15,16]. Nevertheless, the weak mechanical strength of pure chitosan aerogels limits their adaptability in many application environments. Furthermore, the flame retardant performance of chitosan aerogels remains inadequate for practical applications due to their predominant composition of carbon, hydrogen, and oxygen [17,18,19]. Consequently, extensive research efforts have been devoted to designing thermally insulating chitosan aerogels with distinctive mechanical properties and robust flame retardancy through various modification strategies [20,21].

The addition of flame retardants is one of the commonly used treatments for aerogel substrates. So far, numerous flame retardants have been developed to enhance the flame retardancy of functional composites. Wang et al. introduced hollow glass microspheres into the porous network of chitosan aerogels, achieving a Class A1 non-combustibility rating [13]. Zhang et al. fabricated a composite aerogel composed of chitosan, phytic acid, and mannitol, which exhibited a limiting oxygen index value of 41.1% [22]. Attapulgite (ATP), a hydrated magnesium aluminum silicate mineral, holds substantial potential as a flame retardant additive, benefiting from its excellent flame resistance, non-toxicity, cost-effectiveness, and environmental sustainability [23]. It is worth noting that the rich hydroxyl groups in ATP facilitate its intimate integration with chitosan aerogels through hydrogen bonding, resulting in composites with superior mechanical strength [24]. For example, Liu et al. [25] incorporated attapulgite into cellulose hydrogels to enhance their mechanical properties. This work demonstrated that the reinforcement mechanism of attapulgite arises from the formation of hydrogen bonds with cellulose chains, thereby strengthening the physical cross-linking within the hydrogel network. Zhu et al. [26] employed ATP to support gelatin-based aerogel skeleton, achieving remarkable mechanical robustness and flame retardant properties. Similarly, Wang et al. [27] successfully prepared robust cellulose/clay composite aerogels, in which multiple interactions among the constituents, such as hydrogen bonding, played a crucial role. The rod-like nanoclay particles acted as structural regulators, enhancing mechanical integrity, supporting the framework, and guiding internal alignment. The combination of biomass and mineral synergistically imparts hybrid aerogels with outstanding mechanical strength and effective thermal management capabilities [28,29].

In this study, the chitosan aerogel served as a flexible skeleton, which was combined with ATP through hydrogen bonding interactions between the components. Owing to the excellent interface compatibility, attapulgite effectively enhanced the mechanical strength. Moreover, ATP contributed to a significant advancement in fire safety. The resulting composite aerogel (CS-SATP30%) exhibited impressive mechanical performance (a compressive strength of 633.15 kPa at 80% strain), excellent flame retardancy (an LOI of 34%), and distinguished thermal insulation (0.0343 W/(m·K)), exploiting an ideal approach for the fabrication of fire-safe thermal insulation aerogels [30,31].

## 2. Materials and Methods

### 2.1. Materials

Chitosan (CS) with a degree of deacetylation ≥ 95% and viscosity 100–200 mPa·s was purchased from Shanghai Macklin Biochemical Co., Ltd. (Shanghai, China). ATP (Mg_5_Si_8_O_20_(OH)_2_ (OH_2_)_4_⋅4H_2_O, 500 mesh) was supplied by Changzhou Dingbang New Material Technology Co., Ltd., (Changzhou, China). Concentrated sulfuric acid (H_2_SO_4_, analytical grade, AR) was obtained from Nanjing Chemical Reagent Co., Ltd. (Nanjing, China). Acetic acid (analytical grade, AR) was purchased from Sinopharm Chemical Reagent Co., Ltd. (Shanghai, China). All other reagents were of analytical grade, and deionized water (DI) was prepared using a laboratory ultrapure water system.

### 2.2. Methods

#### 2.2.1. Acid Activation of Attapulgite

The acid activation of ATP was performed following the procedure described in previous work [32]. Five grams of dry ATP was dispersed in 50 mL of 2.0 M sulfuric acid aqueous solution and stirred at 60 °C for 2 h, followed by filtration to separate the solid. The solid was washed with DI until the filtrate became neutral. Finally, the acid-activated attapulgite (SATP) was obtained by drying in a vacuum oven at 80 °C for 12 h. Acid treatment can modify the structure of attapulgite by affecting the dissolution and reconstruction of its silicate tetrahedra and alumina octahedra. This process, influenced by parameters such as acid type, concentration, treatment time, and temperature, optimizes the pore structure and surface active groups of the clay [33].

#### 2.2.2. Preparation of CS-SATP Composite Aerogels

**Figure 1:**
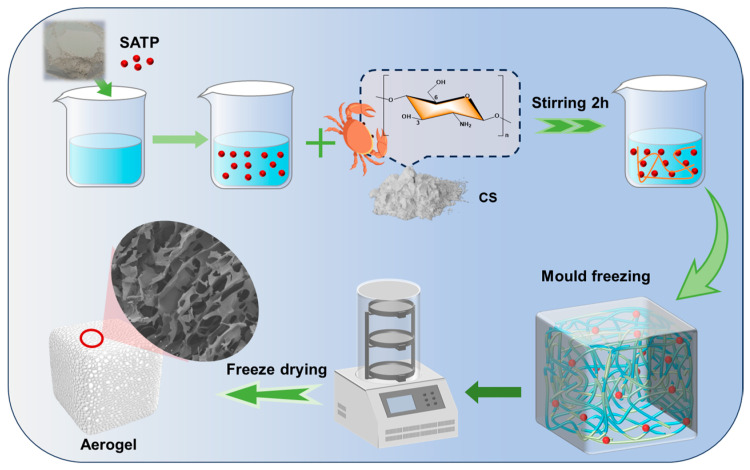
Schematic diagram of the preparation process of CS-SATP composite aerogel.

First, acid-activated attapulgite was dispersed in 100 mL of deionized water by stirring for 10 min, followed by ultrasonication for 15 min to achieve a uniform dispersion. Subsequently, 1 mL of acetic acid was added and mixed thoroughly by stirring, followed by the addition of 1.6 g of chitosan powder. The mixture was stirred continuously at 1000 rpm for 3 h. After removing air bubbles via sonication, the CS-SATP mixed gel was poured into a silicone mold. The resulting gel was frozen and solidified in a low-temperature freezer at −18 °C for 12 h, followed by vacuum drying for 48 h to obtain the composite aerogel material. By varying the SATP content, pure CS aerogel, CS-SATP10%, CS-SATP20%, and CS-SATP30% composite aerogels were fabricated (formulations are listed in Table 1, and the process flow is illustrated in Figure 1). Detailed preparation steps are provided in the Supporting Information.

### 2.3. Characterization Methods

The structure, morphology, thermal stability, flame retardancy, thermal insulation performance, and mechanical properties of the samples were characterized by scanning electron microscopy (SEM), coupled with energy-dispersive X-ray spectroscopy (EDS), Fourier transform infrared spectroscopy (FT-IR), thermogravimetric analysis (TGA), limiting oxygen index (LOI), vertical burning tests (UL-94), cone calorimetry (CONE), Raman spectroscopy (Raman), thermal conductivity and insulation tests, mechanical property tests, and density and porosity measurements. Detailed procedures are provided in the Supporting Information.

## 3. Results

### 3.1. Structural and Morphological Characterization

**Figure 2:**
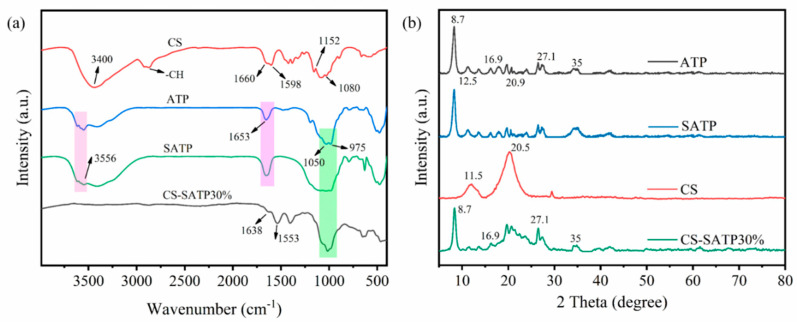
FT-IR spectra of CS, ATP, SATP, and CS-SATP30% (**a**) and XRD patterns (**b**).

FT-IR and SEM were employed to investigate the chemical structure and micromorphology of the prepared materials. As observed in the FT-IR spectra of Figure 2a, ATP and SATP exhibit highly similar characteristic peaks. Specifically, the peak at 3556 cm^−1^ corresponds to the stretching vibrations and out-of-plane bending vibrations of water bound to Mg and Al octahedrons. The absorption peak at 1653 cm^−1^ is associated with coordinated water in ATP, while the characteristic peaks at 1050 cm^−1^ and 975 cm^−1^ are attributed to the Si-O stretching vibrations of ATP [32,34]. Notably, none of these peaks disappeared after acid activation, indicating the preservation of the basic silicate structure. Pure CS exhibits a broad and intense absorption peak in the wavenumber range of 3400 cm^−1^, which arises from the overlapping stretching vibrations of -OH and -NH_2_ groups in its molecular structure. Their synergistic effect results in spectral band broadening and the formation of multiple absorption characteristics. The -CH_2_ stretching vibration can be observed at 2968 cm^−1^ [35]. A characteristic peak corresponding to amide I was observed at 1660 cm^−1^ [36]; a characteristic peak corresponding to the in-plane bending vibration of N-H in amino groups was observed at 1598 cm^−1^. The absorption peaks at 1152 cm^−1^ and 1080 cm^−1^ are assigned to the asymmetric stretching vibration of the C-O-C bond and the symmetric stretching vibration of the C-O bond, respectively [37]. The composite aerogels still retain the characteristic peaks of Si-O, whereas the intensities of the bands associated with amide I, amide II, and hydroxyl groups in CS are moderately reduced. This observation is inferred to stem from the formation of hydrogen bonding interactions at the interface between SATP and CS—for instance, hydrogen bonds are formed between the -OH and -NH_2_ groups of CS and the surface -OH groups of SATP—indicating the successful preparation of the composite material [38].

The effect of acid treatment on the crystal structure of ATP was examined by XRD (Figure 2b). The diffraction pattern of pure ATP shows characteristic peaks at 2θ = 8.6°, 20.9°, 27.1°, and 35°, corresponding to the (110), (040), (400), and (440) crystal planes, respectively. Among these, the strongest diffraction peak at 2θ = 8.6° corresponds to the basic interlayer spacing of ATP’s layered structure, indicating high crystallinity. Additionally, the peaks at 2θ = 12.5° and 16.9° are attributed to the Si-O-Si microcrystalline layers in ATP [39]. The XRD patterns reveal that the crystal structure of ATP is retained after acid activation, which is consistent with the FT-IR results. The characteristic diffraction peaks of CS at 2θ = 11.5° and 20.5° are clearly observable [40]. However, in the composite aerogels, the peak intensity at 11.5° is significantly diminished, indicating a decrease in the crystallinity of CS. This is likely due to hydrogen bonding between the dispersed SATP and the –NH_2_/–OH groups of CS, which disrupts the ordered packing of CS chains [41], a conclusion consistent with the FT-IR analysis.

**Figure 3:**
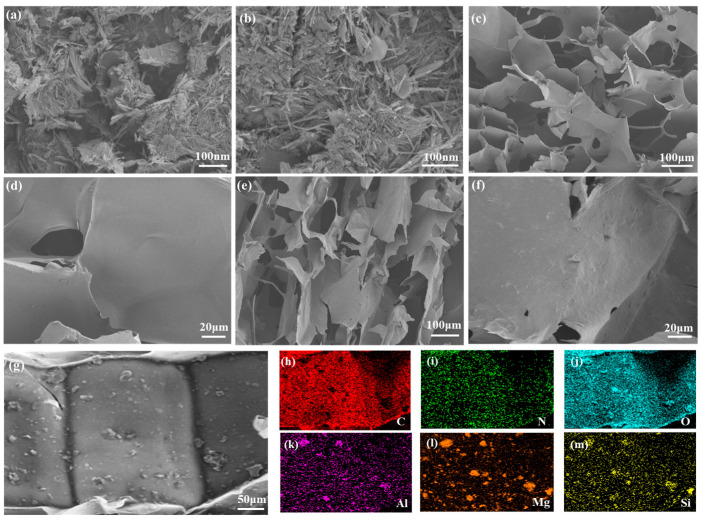
SEM images of ATP (**a**), SATP (**b**), CS (**c**,**d**), and CS-SATP30% (**e**–**g**) and results of elemental distribution of C, N, O, Al, Mg, and Si (**h**–**m**) for CS-SATP30% (**g**).

The microstructure of the materials was further observed via SEM. As shown in Figure 3, pure ATP exhibits a needle-like rod structure. After acid activation, the size of SATP is slightly reduced—this is attributed to the etching effect of acidic conditions on the octahedral and tetrahedral structures of ATP, resulting in shortened lengths while the basic morphology remains intact [36]. As observed in Figure 3c–e, all prepared aerogels exhibit a 3D porous network structure. The pure CS aerogel displays a smooth and flat surface morphology, while micron-scale granular protrusions are visible on the surface of the composite aerogels (Figure 3d–f), a phenomenon attributed to the uniform dispersion of the flame retardant SATP on the aerogel surface. EDS elemental mapping of the composites confirms the homogeneous distribution of C, N, O, Al, Mg, and Si, providing direct evidence for the successful incorporation of the flame retardant filler into the aerogel matrix.

### 3.2. Thermal Stability Analysis

**Figure 4:**
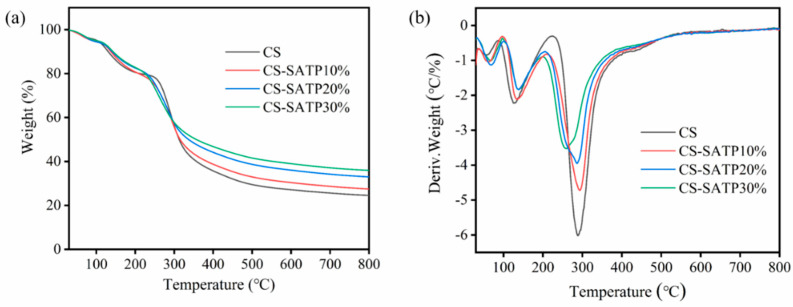
TG (**a**) and DTG (**b**) curves of different composite aerogels under N_2_ atmosphere.

TGA was employed to evaluate the thermal stability of the materials [42]. Figure 4 presents the TG and DTG curves of the materials heated from 30 °C to 800 °C under a nitrogen atmosphere, with specific data summarized in Table 2. The pyrolysis process of all CS-based aerogels in nitrogen consists of two main weight loss stages. The initial mass loss below 200 °C is primarily due to the evaporation of physically absorbed water, which is facilitated by the abundant polar groups (e.g., hydroxyl group and amino group) in chitosan that readily retain moisture via hydrogen bonding. A major, sharp weight loss occurs between 200 °C and 400 °C, corresponding to the thermal decomposition of the polymer’s three-dimensional cross-linked network, including chain scission and backbone degradation [43]. With the incorporation of the flame retardant SATP, the residual char yield of the material increased from 24.41% to 35.67%. Compared with recently reported bio-based aerogels [44,45,46], the resultant CS-SATP30% aerogels demonstrated satisfactory flame retardant properties. This enhancement is attributed to the ability of ATP to promote the formation of a more stable and cohesive char layer at high temperatures, thereby improving carbonization efficiency.

Additionally, the temperature corresponding to the maximum weight loss rate (T_d max_) of the CS-SATP30% composite aerogel is slightly lower than that of the pure CS aerogel. This can be explained by several potential mechanisms. First, SATP, a fibrous clay mineral with a high specific surface area and reactive hydroxyl groups, interacts with CS through hydrogen bonding and electrostatic forces, disrupting the natural crystalline structure of CS and reducing its thermal resistance as well as the energy required for decomposition. Second, functioning as a nanofiller, SATP forms nanoscale interfaces within the CS matrix, where defects or voids generated by its dispersion act as initiation sites for thermal degradation, accelerating decomposition at relatively low temperatures. Finally, structural and adsorbed water within the SATP channels can form hydrogen bonds with the hydroxyl and amino groups of CS, and the catalytic hydrolysis reaction during heating further lowers the decomposition temperature.

### 3.3. Flame Retardancy Analysis

**Figure 5:**
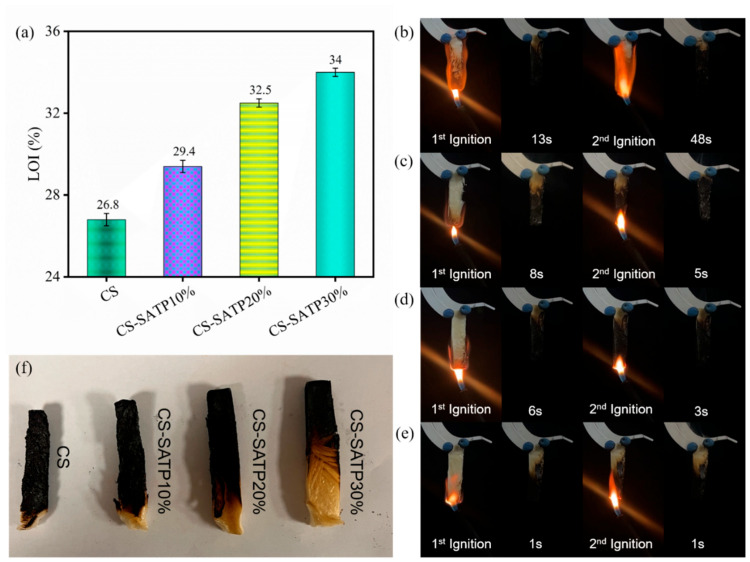
Limiting oxygen index of different composite aerogels (**a**), digital video screenshots of vertical combustion tests of low-temperature gels CS (**b**), CS-SATP10% (**c**), CS-SATP20% (**d**), CS-SATP30% (**e**), comparison of residual carbon after combustion of different aerogels (**f**).

The flame retardant performance of the aerogels was evaluated based on the LOI and UL-94 test results, with detailed data summarized in Table 3. As shown in Figure 5a, the pure CS aerogel exhibited an LOI value of 26.8% due to the presence of nitrogen in its molecular structure. All composite aerogels exhibited higher LOI values, exceeding 27%. The CS-SATP30% composite achieved an LOI of 34.0%, representing a 26.8% increase over pure CS and indicating significantly enhanced flame retardancy. Visual observations of combustion behavior revealed that the pristine CS aerogel ignited immediately upon exposure to fire, exhibited obvious smoldering after re-ignition, and showed a substantial reduction in residual dimensions compared to the modified aerogels (Figure 5). In contrast, the CS-SATP30% composite effectively suppressed smoldering, with a total after-flame time of less than 2 s over two ignition applications, meeting the criteria for a V-0 rating according to the UL-94 standard. These results confirm the effectiveness of SATP in improving the flame resistance of chitosan aerogels.

**Figure 6:**
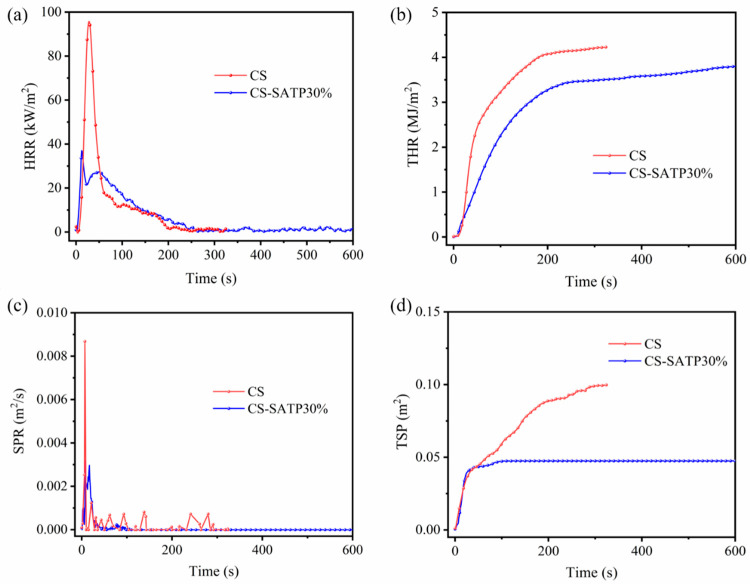
HRR curves for CS and CS-SATP 30% (**a**), THR curves (**b**), SPR curves (**c**), and TSP curves (**d**).

Cone calorimetry tests were conducted to further investigate the combustion behavior under simulated real-fire conditions. Figure 6 presents the time-dependent changes in HRR and THR, with key parameters summarized in Table 3, including TTI, PHRR, FIGRA, and TSP. The pure CS aerogel exhibited a TTI of 7 s, a PHRR of 98.78 kW/m^2^, and a THR of 4.22 MJ/m^2^. In contrast, after the incorporation of SATP, the THR and PHRR of the composite aerogels decreased to 3.83 MJ/m^2^ and 37.00 kW/m^2^, representing a reduction of 9.2% and 62.5% compared to the pure CS aerogel, respectively. Owing to the formation of a dense char layer by SATP during combustion, a significant decreasing trend was also observed in the TSP.

On the other hand, as a key parameter for evaluating the fire risk of materials, FIGRA is characterized as the ratio of PHRR to TpHRR; a lower FIGRA value indicates higher fire safety of the material [47]. As indicated by the data in Table 4, the fire growth rate index (FIGRA) of CS-SATP30% decreased from 3.53 kW/(m^2^·s) to 2.85 kW/(m^2^·s), demonstrating a lower fire risk rating. Based on the comprehensive analysis above, the incorporation of the flame retardant SATP effectively enhances the fire resistance of CS-based aerogels and plays a positive role in reducing the material’s fire risk.

### 3.4. Flame Retardant Mechanism Analysis

**Figure 7:**
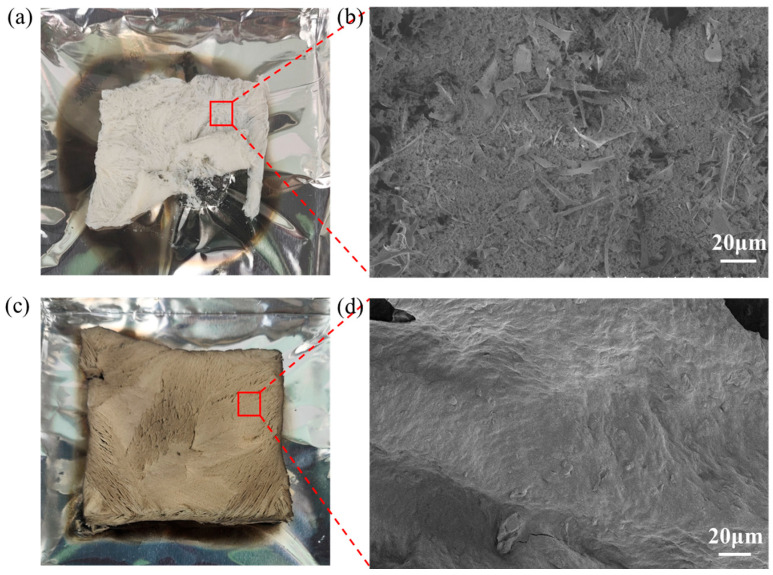
Digital photographs of CS and CS-SATP30% residual charcoal (**a**,**c**) and SEM images of residual charcoal (**b**,**d**).

To investigate the flame retardant mechanism of the composite aerogels, the characteristics of the char residues were analyzed from both macroscopic and microscopic perspectives using digital images and SEM micrographs (Figure 7). After combustion, the CS aerogel exhibited significant volume shrinkage and formed white fluffy residues. SEM observations revealed that its char residue had a loose and fragmented structure. In contrast, the aerogels incorporated with SATP retained a relatively intact morphology after combustion. As shown in Figure 7d, a dense and coherent char layer was formed on their surface. This continuous char layer can effectively act as a physical barrier, impeding direct contact between the underlying material and the flame, inhibiting the transfer of oxygen and heat to the interior, and thus suppressing further combustion [48].

**Figure 8:**
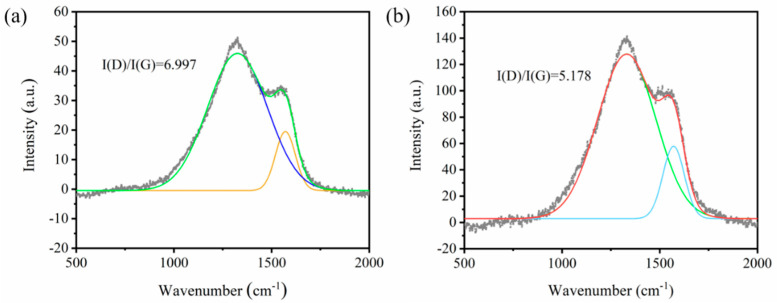
Raman spectra of residual charcoal for (**a**) CS and (**b**) CS-SATP30%.

Raman spectroscopy was further employed to elucidate the structural characteristics of the char layers. As observed in Figure 8, the characteristic peaks at 1340 cm^−1^ and 1580 cm^−1^ correspond to the D-band and G-band of carbonaceous materials, respectively [49,50]. The relative content of graphitic carbon in the materials can be quantitatively evaluated by calculating the integrated intensity ratio of the two bands (I*_D_*/I*_G_*). Generally, a lower I*_D_*/I*_G_* ratio indicates a higher proportion of ordered graphitic structures within the material, corresponding to a more compact char layer with superior thermal stability [51]. The test results show that I*_D_*/I*_G_* of the characteristic peaks for the pristine CS-based aerogel (without flame retardant) is 6.997, while the ratio decreases to only 5.178 after the incorporation of SATP. This change indicates that SATP can significantly promote the graphitization transition during the carbonization of CS-based aerogels, which is consistent with the morphological characteristics of the char residues observed via SEM.

**Figure 9:**
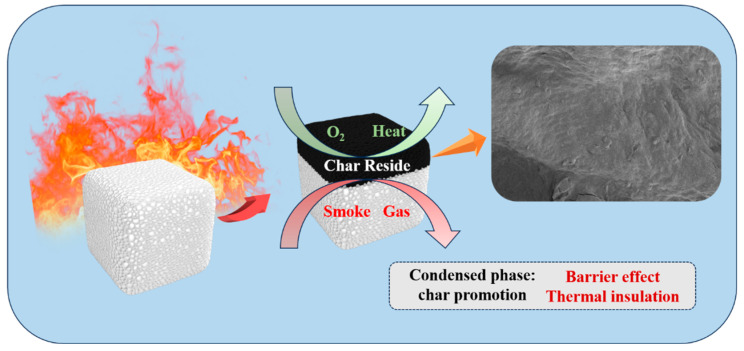
Flame retardant mechanism of CS-SATP30% aerogel during combustion.

Based on the CONE, SEM, and Raman spectroscopy results, a schematic diagram illustrating the flame retardant mechanism of the CS-SATP30% composite aerogel was constructed (Figure 9). The analysis reveals that the flame retardant mechanism of the composite aerogel is centered on condensed-phase flame retardancy. Upon thermal decomposition under high-temperature conditions, SATP generates layered or coked products (e.g., SiO_2_, MgO), which form a dense ceramified protective layer on the material surface. This layer effectively blocks the diffusion of oxygen, heat, and combustible volatiles, thereby inhibiting flame propagation; meanwhile, metal ions (e.g., Mg^2+^, Al^3+^) in SATP can catalyze the formation of a more stable char layer from the matrix at elevated temperatures, enhancing carbonization efficiency [52,53]. The dense char layer further hinders heat and oxygen transfer, slowing down the material’s combustion rate [54].

### 3.5. Thermal Insulation Performance Analysis

**Figure 10:**
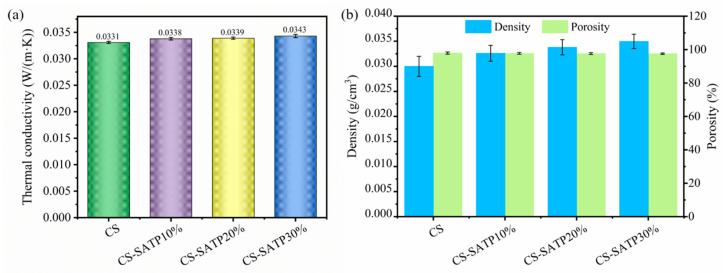
Thermal conductivity (**a**) and density and porosity (**b**) of different composite aerogels.

The thermal insulation performance of the materials was evaluated based on their thermal conductivity, where a lower value indicates superior insulating efficiency. Figure 10 presents the performance metrics of different composite aerogels, including thermal conductivity, density, and porosity. The thermal conductivity of the aerogels slightly increases from 0.0331 W/(m·K) (pristine CS) to 0.0343 W/(m·K) (CS-SATP30%). This minimal change has a negligible impact on the overall insulation performance. According to heat transfer principles, the total thermal conductivity of aerogels is jointly determined by thermal convection, gas conduction, solid conduction, and thermal radiation. Under constant temperature and pressure conditions, the influence of thermal radiation can be approximately neglected. Porosity is a critical factor affecting thermal conductivity. Generally, higher porosity corresponds to lower thermal conductivity and better thermal insulation performance [53]. The pristine CS aerogel exhibits excellent thermal insulation capability due to its high porosity of 97.95%. However, with the incorporation of the flame retardant SATP, the density of the composite aerogels increases slightly, resulting in a modest decrease in porosity. Although the incorporation of SATP slightly increased the density and reduced the porosity of the composites, the change was insufficient to significantly alter their thermal conductivity.

**Figure 11:**
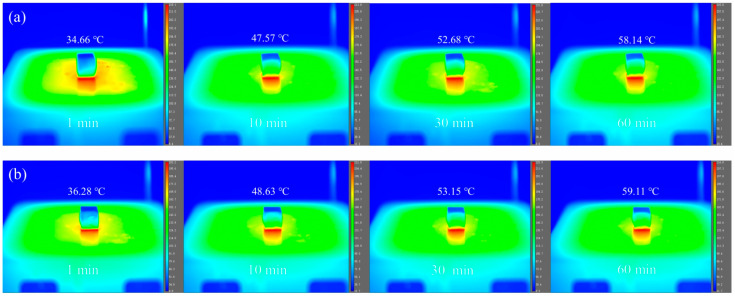
Infrared thermography of CS (**a**) and CS-SATP30% (**b**) on a heated table at 200 °C.

To further investigate the heat transfer characteristics and thermal insulation performance of the aerogels, the samples were placed on a heating platform maintained at 200 °C, and their temperature evolution was continuously monitored for 60 minutes using an infrared thermal imaging system. As observed in Figure 11, the temperature of the aerogels’ upper surfaces gradually increased over time. After 60 minutes of heating, the upper surface temperature of the CS-SATP30% composite aerogel was only 0.97 °C higher than that of the pristine CS aerogel. This result indicates that the prepared composite aerogels possess excellent thermal insulation performance, which is consistent with the measured thermal conductivity data.

### 3.6. Mechanical Property Analysis

**Figure 12:**
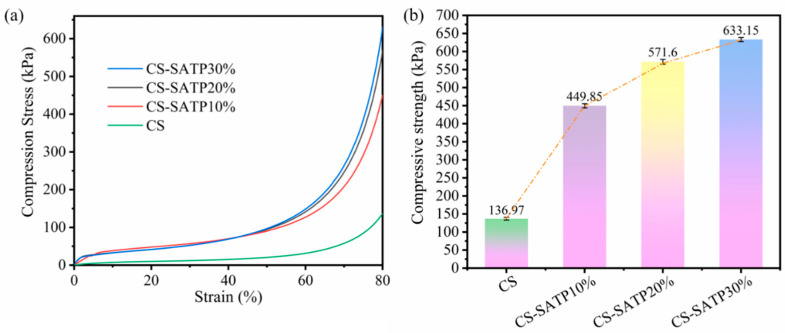
Compressive stress–strain curves (**a**) and compressive strength (**b**) of different composite aerogels.

The mechanical properties of the CS and CS-SATP composite aerogels were assessed by compression tests up to 80% strain. The stress–strain curves of the aerogels clearly reflect three typical stages during the compression process: a linear elastic deformation stage within the low-strain range, a nonlinear plastic plateau stage in the medium-strain region, and a densification stage at the high-strain interval [54]. As illustrated in Figure 12, the incorporation of the flame retardant SATP significantly enhances the compressive strength of the composite aerogels, with all tested samples capable of withstanding 80% compressive strain without structural failure. SATP is composed of a large number of rod-like or acicular nanocrystals with layered and chain-like structures, which effectively reinforce the aerogel skeleton, leading to improved mechanical performance.

## 4. Conclusions

In summary, CS-SATP composite aerogels with robust mechanical strength, excellent flame retardancy, and effective thermal insulation were successfully fabricated via a simple physical blending and freeze-drying method. Multi-scale structural characterization confirmed that SATP formed a stable hydrogen bonding network with the CS matrix, constructing a uniform 3D porous skeleton that significantly improved the mechanical brittleness of pure CS aerogels; CS-SATP30% exhibited a compressive strength of 633.15 kPa at 80% strain. For flame retardancy, SATP endowed the aerogels with superior fire safety, achieving an LOI of 34% and a UL-94 V-0 rating. Mechanistic studies revealed that the SATP-dominated condensed-phase flame retardant mechanism promoted dense ceramifiable char layer formation, effectively blocking heat and oxygen transfer and suppressing smoke release. Moreover, despite a slight density increase, the composite aerogels maintained low thermal conductivity (0.0331–0.0343 W/(m·K)), showing great potential for high-temperature thermal insulation. This work provides a feasible approach for developing high-performance, eco-friendly bio-based thermal management materials.

## Figures and Tables

**Table 1:** Formula ingredients of different composite aerogels.

Sample	CS (g)	ATP (g)	Deionized Water (mL)
CS	1.6	-	100
CS-SATP10%	1.6	0.16	100
CS-SATP20%	1.6	0.32	100
CS-SATP30%	1.6	0.48	100

**Table 2:** Thermogravimetric parameters of different composite aerogels.

Sample	T_d10%_ (°C)	T_d max_ (°C)	C_y800_ (%)
CS	130.40	288.34	24.41
CS-SATP10%	136.53	290.95	27.17
CS-SATP20%	140.23	286.15	32.77
CS-SATP30%	142.98	256.21	35.67

**Table 3:** Ultimate oxygen index and UL-94 test results for different composite aerogels.

Sample	LOI (%)	Dripping	t_1_/t_2_ (s)	UL-94
CS	26.8 ± 0.3	No	13/48	NR
CS-SATP10%	29.4 ± 0.3	No	8/5	V-0
CS-SATP20%	32.5 ± 0.2	No	6/3	V-0
CS-SATP30%	34.0 ± 0.2	No	1/1	V-0

**Table 4:** Cone calorimetry data for CS and CS-SATP30%.

Sample	TTI (s)	THR (MJ/m^2^)	PHRR (kW/m^2^)	TpHRR (s)	SPR (m^2^/s)	TSP (m^2^)	FIGRA (kW/(m^2^·s))
CS	7	4.22	98.78	28	0.0868	0.103	3.53
CS-SATP30%	3	3.83	37.00	13	0.0297	0.047	2.85

## Data Availability

The data presented in this study are available upon request from the corresponding author due to privacy concerns.

## Supplementary Materials

The following supporting information can be downloaded at https://www.mdpi.com/article/10.3390/polym18010098/s1, Table S1: Thermogravimetric parameters of different composite aerogels, Table S2: Ultimate oxygen index and UL-94 test results for different composite; Figure S1: FT-IR spectra of CS, ATP, SATP and CS-SATP30% (a) and XRD patterns (b), Figure S2: SEM images of ATP (a), SATP (b), CS (c,d), and CS-ATP30% (e–g), results of elemental distribution of C, N, O, Al, Mg, and Si (h–m) for CS-ATP30% (g).

## Abbreviations

The following abbreviations are used in this manuscript:

CS	Chitosan
ATP	Attapulgite
SATP	Acidified attapulgite
THR	Total heat release
TTI	Time to ignition
FIGRA	Fire growth rate index
PHRR	Peak heat release rate
TpHRR	Time to peak heat release rate
LOI	Limiting oxygen index
DI	Deionized water
SEM	Electron microscopy
XRD	X-ray diffraction
EDS	Energy-dispersive spectroscopy
FT-IR	Fourier transform infrared spectroscopy
TGA	Thermogravimetric analysis
UL-94	Vertical burning test
CONE	Cone calorimetry
Raman	Raman spectroscopy
