# Supplementary material for: Facile Fabrication of Attapulgite-Modified Chitosan Composite Aerogels with Enhanced Mechanical Strength and Flame Retardancy for Thermal Insulation
Source: Polymers (Basel). 2025 Dec 29;18(1):98. doi: 10.3390/polym18010098 (PMC12787402; doi:10.3390/polym18010098)
Supplement: Supplementary file 1 [file polymers-18-00098-s001.zip › polymers-4038268-supplementary.pdf]

polymers

MDPI

Supplementary information for:

Facile Fabrication of Attapulgite-Modified Chitosan Composite Aerogels with Enhanced Mechanical Strength and Flame Retardancy for Thermal Insulation

Siyuan Cheng [1,2], Yuwen Shao [1], Meisi Chen [1], Chenfei Wang [1], Xinbao Zhu [1,2], Xiongfei Zhang [1,*] and Bo Fu [1,*]

[1] Jiangsu Co-Innovation Center of Efficient Processing and Utilization of Forest Resources, Huaian Industrial Research Institute, Gas Separation Engineering Technology Research Center, College of Chemical Engineering, Nanjing Forestry University, Nanjing 210037, China
[2] Synorm Chemical Co., Ltd., No. 16 Zijin Road, Circular Economy Zone, Huizhou District, Huangshan 245900, China
* Correspondence: zxf1990@njfu.edu.cn (X.Z.); fubo@njfu.edu.cn (B.F.)

I Synthesis of Flame Retardant SATP

First, 5 g of dried ATP was dispersed in 50 mL of 2.0 M sulfuric acid aqueous solution, followed by stirring at 60 °C for 2 h. After the reaction, the solid product was separated by filtration and repeatedly washed with deionized water until the filtrate became neutral. Finally, SATP was dried in a vacuum oven at 80 °C for 12 h to obtain the target modified product.

II Synthesis of CS-SATP Composite Aerogel

First, SATP was dispersed in 100 mL of deionized water, stirred for 10 min, and then ultrasonicated for 15 min to achieve uniform dispersion. After adding 1 mL of acetic acid and mixing thoroughly, 1.6 g of CS powder was added, followed by stirring at 1000 rpm for 3 h. After removing air bubbles via ultrasonication, the CS-SATP mixed gel was poured into a silicone mold. The preprocessed sample was freeze-solidified in a low-temperature freezer at -18 °C for 12 h, and subsequently transferred to a freeze-drying apparatus for drying under vacuum for 48 h to finally obtain the composite aerogel material. Following the aforementioned preparation process, CS-SATP composite aerogel samples with different mass ratios were successfully fabricated by adjusting the addition proportion of the flame retardant.

III Characterization methods

(1) Nuclear magnetic resonance spectroscopy analysis: Nuclear magnetic resonance spectrometer, AV600, BRUKER, Switzerland, using dimethyl sulfoxide (DMSO) as solvent for nuclear magnetic resonance hydrogen spectrum analysis.

(2) Fourier transform infrared spectroscopy test: The sample was characterized by FT-IR using a Brucker VERTEX 80V spectrometer in attenuated total reflectance (ATR) mode. The scanning wave number range was set to 4000-500 cm-1, the number of scans was 32, and the resolution was 4 cm^{-1}.

(3) Scanning electron microscopy and energy spectrum analysis: The microscopic morphology of the sample before and after combustion was observed using a Regulus

8100 field emission scanning electron microscope (FE-SEM) at an accelerating voltage of 3 kV, and EDS energy spectrum was collected on the local area.

(4) Thermogravimetric analysis test: The thermal stability of the sample was analyzed using a NETZSCH TG 209 F thermogravimetric analyzer in a 50 mL/min nitrogen protective atmosphere at a heating rate of 10 K/min. The temperature range was set to 30-800 ℃.

(5) Limiting oxygen index determination: According to the GB/T 2406.2-2009 test standard, aerogel specimens with a size of 100 mm × 10 mm × 10 mm were tested using a JF-3 oxygen index tester. The test was repeated three times in parallel and the average value was taken.

(6) Vertical combustion performance test: According to the GB/T 2046.2-2009 standard, a UL-94 vertical combustion test was carried out on a sample with a size of 100 mm × 10 mm × 10 mm.

(7) Cone calorimetry test: Referring to the ISO 5660 international standard, a WK5243-PC cone calorimeter was used to test the combustion performance of a sample of 100 mm × 100 mm × 20 mm at an irradiation intensity of 35 kW/m^2.

(8) Raman spectroscopy analysis: The degree of graphitization of the combustion residual carbon layer was quantitatively characterized by using a DXR532 laser Raman spectrometer using a 532 nm laser line.

(9) Thermal conductivity and insulation performance test: A TC 3000E thermal conductivity meter was used to measure the thermal conductivity of a sample of 30 mm × 30 mm × 6 mm at room temperature (25 ℃) based on the transient hot line method. Simultaneously, the sample was placed on a 200 ℃ heating table surface, and its thermal insulation performance was evaluated using a T1050sc infrared thermal imager.

(10) Mechanical properties test: In accordance with the national standard GB/T 13480-2014, a CMT850 electronic universal testing machine equipped with a 2 kN load cell was used to perform uniaxial compression tests on 20 mm × 20 mm × 20 mm cube specimens at a compression rate of 10 mm/min. The compressive strength and modulus data were recorded when the strain reached 80%. Each group of samples was tested three times and the average value was taken.

(11) Density and porosity test: First, the length, width, and height parameters of the aerogel specimen were measured using a vernier caliper, and its volume V was calculated based on the geometric formula; then, the sample mass m was weighed using an electronic balance with an accuracy of 0.1 mg. Finally, the aerogel density was calculated according to formula (2-1) :

$$\rho = \frac{m}{V} \tag{2-1}$$

the porosity was calculated according to the following equation (2-2):

$$\text{Porosity (\%)} = \left(1-\frac{\rho}{\rho_0}\right) \times 100\% \tag{2-2}$$

Where ρ represents the apparent density of the aerogel, and ρ_0 represents the skeleton density of chitosan. ρ_0 is 1.46 g/cm^3, which is provided by the material supplier.

IV Thermogravimetric Analysis

Table S1. Thermogravimetric parameters of different composite aerogels.

Sample	$T_{d10\%}$ (℃)	$T_{d\,max}$ (℃)	C_{y800} (%)
CS	130.40	288.34	24.41
CS-SATP10%	136.53	290.95	27.17
CS-SATP20%	140.23	286.15	32.77

https://doi.org/10.3390/polym18010098

CS-SATP30%	142.98	256.21	35.67

V Flame retardant performance analysis

Table S2. Ultimate oxygen index and UL-94 test results for different composite aerogels.

Sample	LOI (%)	Dripping	t_1/t_2 (s)	UL-94
CS	26.8 ± 0.3	No	13/48	NR
CS-SATP10%	29.4 ± 0.3	No	8/5	V-0
CS-SATP20%	32.5 ± 0.2	No	6/3	V-0
CS-SATP30%	34.0 ± 0.2	No	1/1	V-0

VI Structural and Morphological Characterization

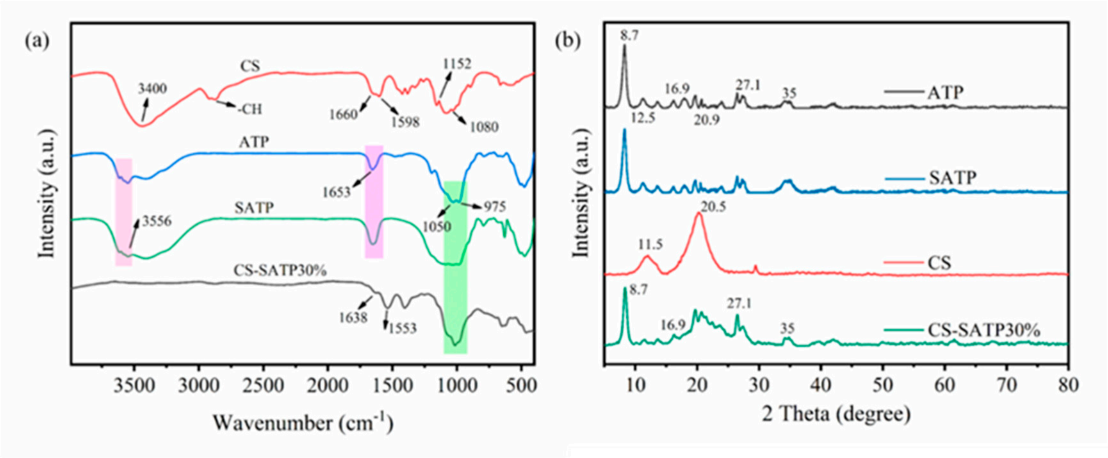

Figure S1. FT-IR spectra of CS, ATP, SATP and CS-SATP30% (a) and XRD patterns (b).

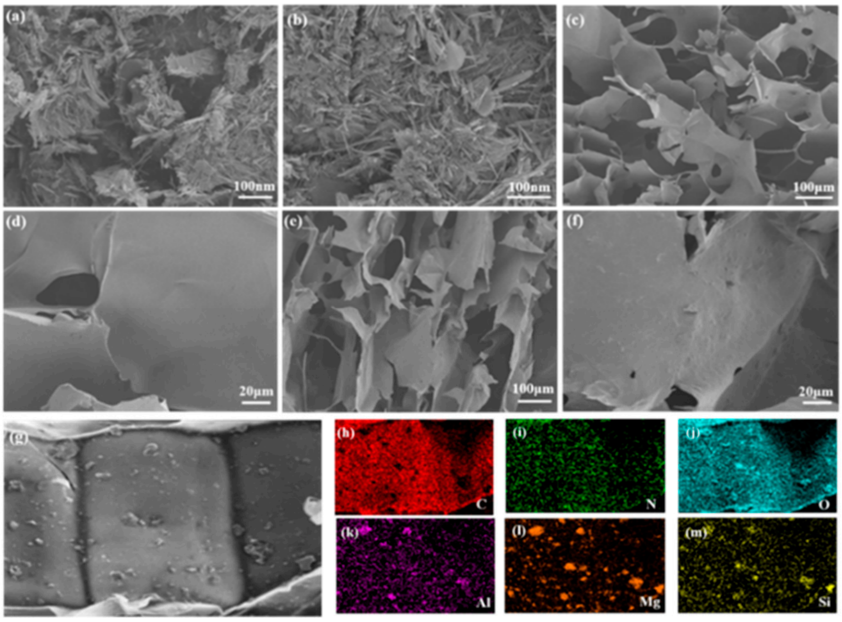

Figure S2. SEM images of ATP (a), SATP (b), CS (c, d), and CS-SATP30% (e-g), results of elemental distribution of C, N, O, Al, Mg, and Si (h-m) for CS-SATP30% (g).

https://doi.org/10.3390/polym18010098
